# Social identification and paranoia

**DOI:** 10.1098/rsos.231961

**Published:** 2024-06-19

**Authors:** A. Greenburgh, L. Zamperetti, V. Bell, N. Raihani

**Affiliations:** ^1^ Department of Health Services and Population Research, Institute of Psychiatry, Psychology and Neuroscience, King’s College London, David Goldberg Centre, De Crespigny Park, London SE5 8AF, UK; ^2^ Department of Experimental Psychology, University College London, London, UK; ^3^ Department of Clinical, Education and Health Psychology, University College London, London, UK; ^4^ School of Psychology, University of Auckland, 23 Symonds Street, Auckland 1010, New Zealand

**Keywords:** social identity, paranoia, cooperation, trust

## Abstract

Paranoia is associated with variation in social behaviour, such as lower inclination to trust others or to behave generously in economic game settings. Such variation may stem, in part, from a reduced tendency to socially identify with others, although previous studies have reported mixed results. We tested whether paranoia involves altered social identification in a pre-registered online study investigating the relationship between a measure of social identification, paranoia, and social behaviours in economic games. We successfully manipulated social identification, but paranoia was associated with slightly increased social identification overall. Neither paranoia nor social identification predicted behaviour in the economic games, and there was no interaction between paranoia and social identification regarding trusting and cooperative behaviours. Our results converge with recent work suggesting that more paranoid individuals may harbour a higher tendency to perceive themselves as having similar beliefs to others. We discuss some key areas for future research to progress understanding in this area.

## 1. Introduction

Paranoia—the belief that others intend to harm you and that this harm will occur [1]—is associated with lower trust and reduced tendency to cooperate in economic games [2–5]. However, the cognitive mechanisms underpinning these patterns are not fully understood. One possibility is that paranoia involves alterations in how individuals relate to others and, specifically, the extent to which they perceive others to be part of their ‘ingroup’. This mechanism is plausible because cooperative and trusting behaviours are known to vary with group membership and social identity [6,7]. Individuals perceive themselves as less similar to outgroup than to ingroup individuals [6,8], and this can manifest as liking outgroup members less than ingroup members [6,9,10], feeling less close to them [11] and empathizing less with them [12]. Other work has shown that people also have higher expectations of being helped by ingroup members and that this expectation of help underpins variation in tendency to behave prosocially towards ingroup members [13]. For example, individuals make less effort on behalf of outgroup members’ goals and share fewer resources with them [6,7,9,14], and even children aged 3–7 exhibit this parochialism [15].

A central project of social psychology has been to understand the processes that determine social identification. Early theories of intergroup psychology posited that ingroup membership may be determined according to members’ similarity [8], shared categorical social group membership [16], interdependent outcomes or ‘common fate’ [17], and the existence of competition over limited resources [10,18]. Coalitional views have recently gained prominence, emphasizing that groups are, at base, relational properties [19] where perception of an agent’s capacity to coordinate with the self and others ultimately determines whether they are considered ingroup members [6]. Common to many of these perspectives is that the broader social context will moderate if and how we relate to others [14,19].

One such contextual factor is the level of social threat present in the environment. Threatening conditions decrease the tendency to classify ambiguous targets as ingroup [20]. For example, individuals are more likely to categorize social stimuli (pictures of faces) as outgroup when these stimuli express threat cues (such as angry facial expressions and high masculinity) and when the individuals themselves harbour chronic beliefs about interpersonal danger [21,22]. Paranoia is associated with an increased perception of social threat in neutral social settings, with more paranoid individuals attributing more hostile intent to neutral interaction partners [23–25]. It may therefore be that paranoia is associated with variation in the tendency to socially identify with others: in other words, more paranoid individuals might be less likely to treat neutral targets as being ingroup members.

Nevertheless, it is not yet clear whether social identification consistently varies with paranoia. While some initial data indicate that self-reported identification with social groups is lower in more paranoid individuals [26,27], this relationship is sometimes absent [26,28]. Experimental studies show that loneliness causally increases paranoia [29,30] and time series data suggest that feeling close to others predicts subsequent low levels of paranoid ideation [31]. However, belonging to a social group can increase paranoia if social interactions with this group are negative [32]. One experimental attempt to manipulate social identification and test the impact of this on paranoia found no effect [26]. Additionally, no study has investigated whether trait paranoia predicts social identification with neutral targets in experimental settings.

We report one online, pre-registered experimental study. We ask whether paranoia involves a higher threshold for identifying with similar others and, if so, whether this reduced tendency to view others as ingroup is associated with reductions in cooperative and trusting behaviour. We investigated (i) whether paranoia is associated with a reduced tendency to identify with explicitly similar others, and (ii) whether social identification and paranoia differentially explain cooperative and trust behaviour in live social interactions.

## 2. Method

Data were collected in June 2021. The project was approved by the University College London (UCL) ethics board (project number 3720/002). Informed consent was obtained from all participants, and the participation was voluntary. In this article, we report all measures, manipulations and exclusions.

### 2.1. Participants

We recruited 195 participants (F = 144, M = 50, did not report = 1) from the Prolific Academic, UK. Participants who did not pass manipulation and comprehension checks were excluded from the final analysis (see §2.2), leaving a sample of 165 participants (F = 126, M = 39). The mean age of this final sample was 38.2 years (s.d. = 12.90, range: 19–81).

### 2.2. Procedure

Data were collected over two phases. In phase 1, all participants completed the revised Green *et al.* Paranoid Thoughts Scale (R-GPTS [33]), and the Beliefs and Values Inventory [34], order randomized between participants. The R-GPTS is an 18-item scale comprising two subscales, where we employed the 10-item *ideas of persecution* subscale as our primary paranoia variable (maximum score = 40), as in Greenburgh *et al.* [35]. The Beliefs and Values Inventory [34] is a 55-item scale where participants are asked to rate their agreement with statements spanning political, scientific, religious, paranormal and moral domains.

One week later, all participants were recalled for phase 2. Here, participants were randomly allocated to control or experimental conditions. In the experimental condition, participants were told that they were assigned to partners who had given similar answers to them in the Beliefs and Values Inventory in phase 1: ‘you will be matched with other participants (your partners) in each game who have similar beliefs and values to you based on at least one question in the questionnaire you completed’. In the control condition, participants were told that they were randomly assigned to partners: ‘you will be paired with a player at random’, and they were also informed that all other players had completed the same questionnaire in phase 1 as they had. All participants were required to complete a manipulation check to test this manipulation, where they were shown the statement, ‘I will be matched with other participants…’ and they were asked to select one of three possible answers, where option 1 was correct in the intervention condition, and option 3 was correct in the control condition: (i) who had similar beliefs and values to me based on at least on question in the questionnaire I completed, (ii) who had different beliefs and values to me based on at least on question in the questionnaire I completed, or (iii) randomly. They were then asked to complete a measure of social identification before taking part in two games.

Social identification was measured by asking participants the degree to which they thought their partners would have similar responses to them on other items overall in the Beliefs and Values Inventory, on a scale of 0–100% (initialized at 50%). Therefore, this question elicited a judgement about the overall similarity to their partner’s beliefs in general rather than on any specific item in the questionnaire. Only items that were not used as a part of the instructional materials were used for this part of the study. Social identification was subsequently coded as an ordered categorical variable with five levels with a minimum of 10 data points per level and, as per our pre-registration, the precedent for this approach in the context of skewed variables can be found in previous work [25,36].

All participants then played a modified Trust Game [37] and a Dictator Game [38] (order randomized between participants). Participants were assigned new partners for each game to minimize spill-over effects and were asked to complete two comprehension check questions for each game. We used a 2 × 2 between-subjects design where the participants were randomly allocated to levels of social identification (control/experimental) and task order (Trust Game first/Dictator Game first).

In the Trust Game, participants took the role of *trustor*. Here, participants begun with an endowment of £0.50 and their partner begun with £1.00. Participants could choose to enter or avoid the interaction with their partner. If they avoided the interaction, their pay-off was £0.50. If they chose to enter the interaction, their partner’s choice was enacted: to either send or take £0.25 to/from the participant. Trust was therefore operationalized as a binary variable: enter or avoid the interaction.

In the Dictator Game, participants played as the *dictator*. They were given an endowment of £0.55 and were asked to choose how much to send to their partner from £0 to £0.55 in £0.05 increments. The amount participants sent to their partner is hereafter referred to as ‘cooperation’. The Dictator Game is routinely used as a measure of cooperation [39,40] as it allows the measurement of cooperative behaviour in the absence of strategic concerns [24]. In line with our pre-registration, cooperation was coded as an ordered categorical variable with five levels with a minimum of 10 data points per level.

Finally, participants played a Trust Game in the role of the *trustee*. Responses in the latter game were not analysed in the current study: they were collected for ex-post matching. Ex-post matching is a technique, whereby partner decisions are pre-collected and stored in order to then be shown to players in game-economic paradigms so that, although partner decisions are not occurring in real time, they do reflect the real choices made by game partners [41].

### 2.3. Pre-registered predictions

Analyses and predictions for the study were pre-registered at https://aspredicted.org/blind.php?x=XQE_OQQ :

- social identification will be higher in experimental than control condition;
- participants scoring higher in paranoia will show lower social identification with ingroup members compared with those scoring lower in paranoia; and
- social identification and paranoia will make unique and shared contributions to trust and cooperative behaviour.

As stated in our pre-registration, we ran a simulated power analysis, which indicated that a sample size of 250 will give 88% power to detect two small-moderate (0.2) effect sizes (e.g. for paranoia and social identification on trust and cooperation output variables) and 90% power to detect a small-moderate interaction effect (between paranoia and condition on social identification).

### 2.4. Statistical analyses

All analyses were performed using R (v. 4.04–4.1.1) and all data and code to reproduce analyses are available at https://osf.io/2twym/?view_only=6f8ca5d3f73243059520e0b25075a8ac.

We conducted cumulative link models (clms) [42] using the R package ‘ordinal’ [43], which allows multiple regression models with ordinal categorical dependent variables to be specified. To determine the parameter estimates, we employed a model selection approach with model averaging for each clm [44] using the R Package MuMIn [45]. Under this approach, a single maximal model is compared with all possible submodels, with those models within two Aikaike information criterion units of the top model forming the top model set. Parameter estimates and confidence intervals (CIs) are obtained by averaging over this top model set, thereby accounting for uncertainty over which is the true ‘best’ model. We report full model averaged effects which are typically more conservative. This information-theoretic approach has many advantages, including not employing arbitrary *p*-values as indicators of significance [46]. In all models, continuous input variables were standardized and binary input variables were centred [47] so that parameter estimates can be interpreted as being on the same scale.

We conducted three clms. The dependent variable for model 1 was social identification (five levels), and input terms were paranoia, condition and the two-way interaction. For model 2, the dependent variable was trust (two levels), and for model 3, the dependent variable was cooperation (five levels). For models 2 and 3, the input terms were paranoia and social identification. Social identification was coded as an ordered categorical variable when it is the output variable and as a standardized continuous variable when it is an input variable. We report one deviation from our pre-registration. We had planned to perform a commonality analysis exploring the unique and shared contributions of paranoia and social identification to trust and cooperative behaviour, respectively (prediction (iii) above). This analysis would determine the degree to which variance in the outcome variable (trust or cooperative behaviour) can be attributed to each predictor variable (paranoia and social identification) uniquely and the proportion of explained variance that is associated with the common effects of these predictors [48]. However, we did not perform this analysis as neither paranoia nor social identification predicted trust or cooperation (see §3).

## 3. Results

All tables and results below present analyses of the data obtained from participants who passed the manipulation and comprehension checks: *n* = 165. The manipulation of social identification was largely successful: only 11.7% of recruited participants were excluded owing to failing the manipulation check. While participants were randomly assigned to control and experimental conditions, the included sample consisted of 67 individuals who participated in the control condition and 98 individuals who participated in the experimental condition. For results including data from non-comprehenders, see the electronic supplementary material.

### 3.1. Paranoia

**Figure 1.**
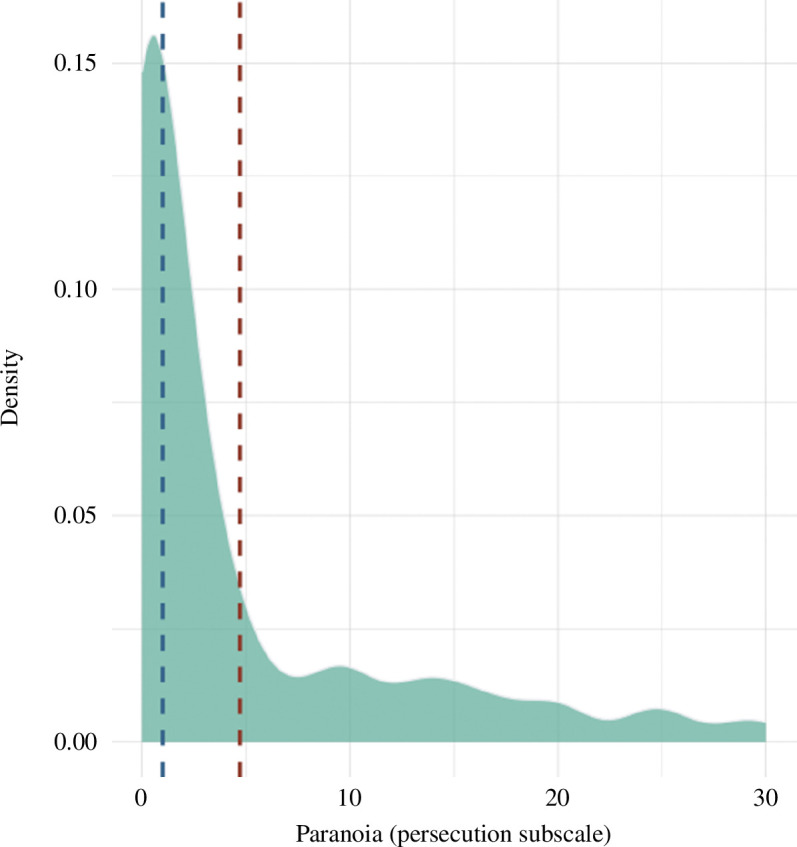
Distribution of paranoia in participants included in the final analyses. Mean paranoia score is represented by the red-dashed line, and the median is represented by the blue-dashed line.

The mean paranoia score of those included in the final analysis was 4.72 (s.d. = 7.24, range = 0−30) out of a possible total of 40. Of this group, 73, 10, 8.5, 6.7 and 1.8% fell in the average, elevated, moderately severe, severe and very severe categories, respectively, as defined by Freeman *et al*. [33]. Very few participants fell in the highest category of paranoia (figure 1): 1.8% scored above the mean for people with persecutory delusions [33]; and the maximum paranoia score (30) was well below the highest possible score of 40.

### 3.2. Social identification

**Figure 2.**
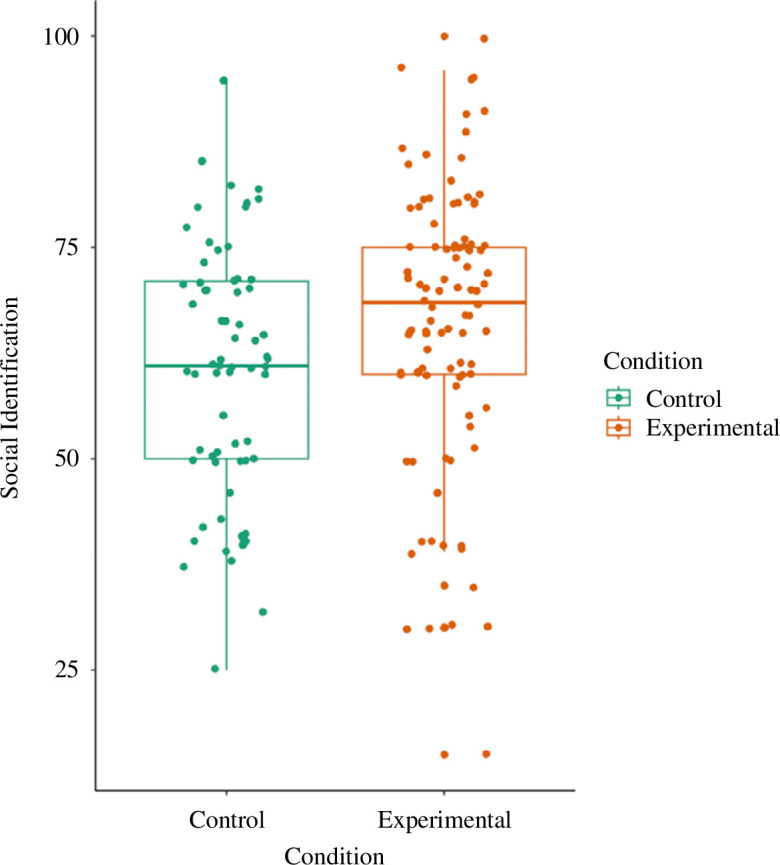
Box plot that shows social identification as a function of condition. Raw data points have been plotted for each condition.

**Figure 3.**
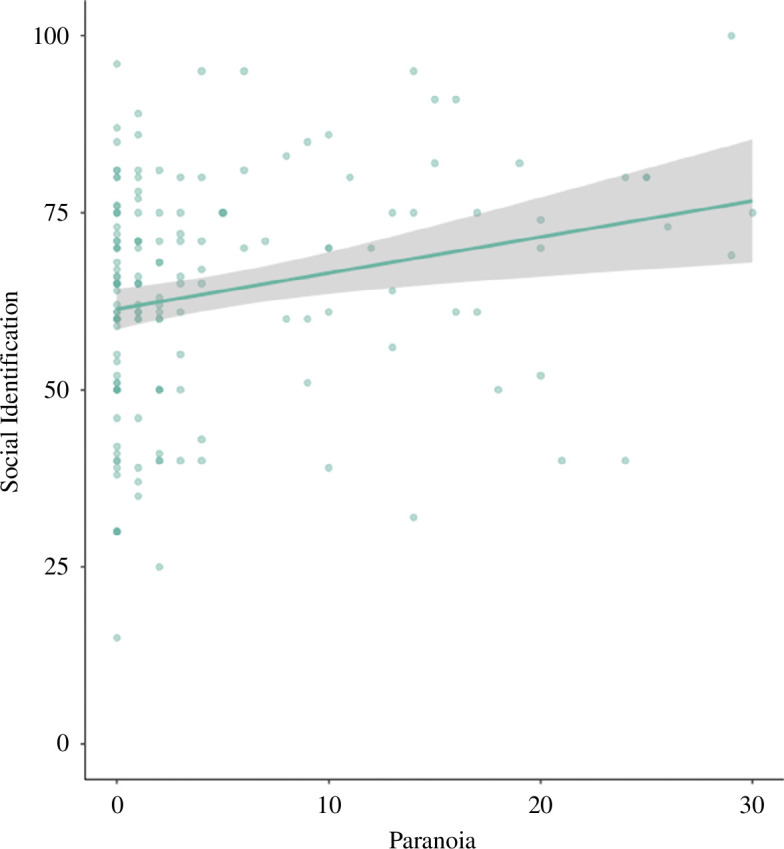
The relationship between paranoia and social identification. Shaded areas represent 95% CIs for predictions of the generalized linear model. Raw data points are also plotted.

As expected, we found increased social identification in the experimental compared with the control condition (table 1; figure 2). Nevertheless, counter to our predictions, paranoia positively predicted social identification overall (table 1; figure 3). There was no interaction effect between paranoia and condition on social identification. When re-running models controlling for age and gender, no qualitative differences were found (see the electronic supplementary material). When including non-comprehenders, we found no associations between paranoia, experimental condition or the two-way interaction and social identification (see the electronic supplementary material).

**Table 1.** Variation in social identification: parameter estimates, unconditional s.e. and 95% CIs for terms included in the top model set. (See the electronic supplementary material for top model set.)

parameter	estimate	unconditional s.e.	95% CI
condition (1 = experimental)	0.62	0.29	(0.05, 1.20)
paranoia	0.37	0.16	(0.05, 0.68)
condition: paranoia	0.33	0.37	(−0.40, 1.07)

### 3.3. Trust and cooperation

In the Trust Game, 69% of participants (*n* = 114) trusted their partner. In the Dictator Game, 83% (*n* = 137) of participants made a positive contribution to their partner; on average, they gave 39.4% of their initial endowment, the modal contribution was £0.25 (*n* = 63) and the contributions ranged from £0 to £0.45 out of a maximum possible donation of £0.55.

Neither paranoia nor social identification predicted trust or cooperation (see models 2 and 3, electronic supplementary material, for top model sets), and this result held even when including those who failed the comprehension and manipulation checks. When re-running models while controlling for age and gender, no qualitative differences were found (see the electronic supplementary material), although this analysis found that men were less trusting than women in the Trust Game (estimate = −0.90, 95% CI = −1.65, −0.14) and that older people were more cooperative in the Dictator Game (estimate = 0.32, 95% CI = 0.03, 0.61). *Post hoc* analyses found that condition (experimental/control) did not predict trust or cooperation, and there was no interaction effect between social identification and paranoia on either of these outcome variables (table 2).

**Table 2.** Variation in trust and cooperation: parameter estimates, unconditional s.e. and 95% CIs for terms included in each top model set for each respective outcome. (See the electronic supplementary material for top model sets.)

outcome	parameter	estimate	unconditional s.e.	95% CI
trust	paranoia	0.08	0.15	(−0.22, 0.37)
	social identification	0.04	0.11	(−0.18, 0.26)
cooperation	paranoia	−0.06	0.14	(−0.34, 0.22)
	social identification	0.05	0.15	(−0.24, 0.35)

## 4. Discussion

In an experimental paradigm, we tested whether (i) paranoia was associated with lower identification with explicitly similar social others, and (ii) the changes in social identification could account for altered cooperative and trust behaviours that typically accompany paranoia. Our manipulation of social identification functioned as expected on those who passed comprehension checks: participants in the experimental condition showed a stronger social identification with others. However, contrary to our pre-registered predictions, social identification in all conditions was increased in those scoring higher in paranoia. In addition, we did not observe any variation in trust and cooperative behaviour in those scoring high in paranoia.

The result that social identification was higher in participants scoring higher in paranoia adds to the inconsistent literature on the association between social identification and paranoia. Discrepancies in results thus far seem to reflect the different measures used, the type of target group identified, and the varying contexts and hypotheses studied. For example, paranoia consistently correlates with some measures of social identification (national identification: lower paranoia associated with greater national identification) but not others (political identification) [26]. Furthermore, despite paranoia being positively associated with social marginalization overall [49], belonging to a social group can increase paranoia where social interactions with this group are negative [32]. Previous work attempting to manipulate social identification and examine its impact on paranoia found no direct effect between social identification and subsequent ratings of paranoia (after excluding a subsample for whom the manipulation was not successful) [26]. The latter study is difficult to compare with ours as these authors were interested in whether higher social identification could reduce subsequent paranoia, whereas we studied whether paranoia involves an altered tendency to socially identify with others. Future research is needed to dissect how such processes may interact.

When focussing on our specific operationalization of social identification—perceived similarity of belief—our results align with recent relevant work. That is, paranoia is associated with a higher perception that others similar to the individual have similar conspiracy beliefs as them [36], and more paranoid individuals believe that members of their social network will share their conspiracy beliefs [50]. Our result extends this work in suggesting that this bias to believe others have similar beliefs to you is not restricted to interactions with explicitly ingroup members but may be present in paranoia with regards to any ambiguous social partner. Future research may extend this work to test if such bias persists with regard to outgroup members. Based on recent findings that advice-taking in individuals scoring higher in paranoia is not altered by group membership of the advisor [51], we might predict that the bias we identified may also apply to outgroup partners.

It is possible that our unexpected result that paranoia was associated with increased social identification in fact reflects variation in other cognitive processes. For example, this result may be underpinned by a reasoning bias previously associated with paranoia: the tendency to form conclusions based on lower degrees of evidence (jumping to conclusions (JTC) bias) [28,52–55]. That is, for the experimental condition where participants were told they were similar to others on one dimension, this reasoning bias could lead participants to overpredict that they would be similar along other dimensions. Indeed, this bias has been associated with other types of belief such as conspiracy thinking [56], although further research is needed to assess whether JTC bias generalizes to social domains. Repeating our current experiment with a non-social control would help to delineate whether paranoia is associated with the overprediction of similarity in general or to social affiliates alone. Alternatively, it may be that participants’ answers reflected how similar to others they *desired* to be rather than how similar they felt. Paranoia is associated with small social networks and low perceived social support [49], and identifying with social groups may reduce paranoia by increasing the individual’s self-esteem [27]. It is therefore possible that people scoring high paranoia who experience isolation desire greater social connection, but their paranoid concerns prevent them from achieving it.

Contrary to our pre-registered prediction, the manipulation of social identification did not impact cooperation and trust. This null result for cooperation in the Dictator Game may be explained by results that indicate ingroup bias in minimal group paradigms is typically lower when there is no mutual interdependence between individuals [57]; however, this does not explain our null result in the Trust Game. Some research indicates that bilateral knowledge of group membership is needed for group-based trust to occur [58,59]. Therefore, participants may have not shown higher trust for ingroup trustees as they may have thought these trustees were unaware of their group membership. Indeed, we did not inform trustees of such information. We note other research has also found no evidence of intergroup discrimination in Trust Games [60]. It is also possible that our manipulation was not sufficiently strong to induce social identification that would elicit intergroup discrimination in cooperative and trust behaviours, as indicated by the marginal effect size of our intervention on social identification.

We also failed to find an association between paranoia and any social behaviour (trust or cooperation). This result contradicts previous research findings of lower cooperation [24] and lower trust [2,3] in individuals scoring high in paranoia from the general population and in patients with psychosis (where paranoia is a common experience). Nevertheless, this literature employing economic games is mixed, particularly with regard to trusting behaviour. For example, studies with individuals with psychosis have produced null results when comparing trusting behaviour in patients and controls [61]; and, a recent study found that paranoia in the general population was not associated with a greater reluctancy to enter interactions with others who have the option to betray the participant across three different experiments [35]. The association between cooperative behaviour and paranoia seems to be more robust [4,5,62]. Indeed, any absence of an association between paranoia and cooperation has been attributed to the inclusion of punishment mechanisms in study design where dictator decisions are therefore strategic rather than purely cooperative [63]. However, as we did not include punishment mechanisms in our Dictator Game in study 2, our null result between paranoia and cooperation remains contrary to reliable findings in previous studies.

We note that, in comparison to a population studied in a meta-analysis of Dictator Game studies [64], participants in our sample were more inclined to cooperate overall and gave higher proportions of their initial endowment. A meta-analysis of 20 813 dictators [64] reported that 63.89% of dictators made a positive contribution to the receivers and, on average, dictators gave 28.35%. In comparison, 83% of participants in our study made a positive contribution to their partner and, on average, they gave 39.4% of their initial endowment. The heightened generosity of our sample may have contributed to our unexpected results.

We note that sampling limitations may have given rise to the results we observed. Primarily, although our initial power analyses indicated that our study would be sufficiently powered to detect the effects of interest, the sample used in our analysis was smaller than intended as some participants failed the comprehension checks. Furthermore, the sample recruited was more highly skewed than we expected, where very few individuals scored above clinical thresholds of paranoia [33], and to a greater extent than in previous studies conducted with online samples [5,23,25,65]. Indeed, our study failed to sample the full range of paranoid thinking. It therefore may have been that our sample did not cover enough of the paranoia spectrum to detect any effects. Additionally, the female skew in the samples may have biased our cooperation results—for example, given gender effects observed in previous studies that females give more than males [24,64]; however, an effect of gender on generosity is not always replicated (e.g. [5]).

We operationalized group membership through cues of explicit similarity. However, group representations are often formed on the basis of more profound behavioural cues [6]. A recent synthesis proposes that groups are relational properties: cognitive representations of groups are formed on the basis of assignments of agents to roles within triadic interactions [19]. Future research involving triads will allow us to examine how forming representations of latent groups might vary in paranoia. Individuals have been shown to copy others who belong to the same latent group within triadic structures, as indicated by choice histories of agents in these triads [66,67]. It may be that paranoia involves more subtle difficulties with forming representations of the self within larger latent groups.

Overall, we successfully manipulated perceived similarity to others in experimental settings to test whether paranoia involves altered social identification mechanisms. Paranoia was associated with higher levels of social identification with others; however, neither this measure of social identification nor paranoia was associated with altered cooperative and trust behaviour.

## Data Availability

All data and code to reproduce analyses are available at [68]. Supplementary material is available online [69].
